# Counseling regarding the care of people with dementia with a focus on §7a SGB XI in Germany: a "gray-shaded" scoping review

**DOI:** 10.1186/s12913-023-09155-7

**Published:** 2023-04-12

**Authors:** Mike Rommerskirch-Manietta, Christina Manietta, Daniel Purwins, Martina Roes

**Affiliations:** 1 Deutsches Zentrum für Neurodegenerative Erkrankungen (DZNE), Site Witten, Witten, Germany; 2 Witten/Herdecke University, Faculty of Health, School of Nursing Science, Witten, Germany

**Keywords:** Support, Family, Caregivers, Health service

## Abstract

**Background:**

Care counseling is an important psychosocial intervention for people with care needs and their relatives and can contribute to maintaining and/or improving a patient’s quality of life and reducing the burden of caregivers. This is especially the case for people with dementia and their relatives, in which the methods of care counseling need to be different than those for individuals with non-dementia related care needs. Furthermore, the counseling content needs to be adjusted to the specific form and stage of dementia. In Germany, every person who receives support per the German Social Law Book XI (SBG XI) can take advantage of care counseling according to §7a SGB XI. To date, there is no systematic overview of counseling services for people with dementia and their relatives related to this specific provision in Germany.

**Methods:**

We conducted a gray-shaded scoping review with a focus on the evaluation of care counseling according to §7a SGB XI for people with dementia and their relatives. For this purpose, we applied five search strategies. We researched (1) national electronic databases, (2) Google, (3) targeted websites, (4) experts, and (5) academic electronic databases. Additionally, for the included gray literature, we conducted backward citation tracking via reference lists and forward citation tracking via Google Scholar for scientific articles. Screening of the identified potentially relevant records was performed independently by two reviewers.

**Results:**

We identified 985 records and included 6 studies in our review. We divided the identified studies into three themes: understanding conceptual dimensions, digitalization of counseling, and understanding the perspective of those being counseled. No studies investigated the perspective and experience of people with dementia and their relatives regarding the counseling service according to §7a SGB XI.

**Conclusions:**

Our results show that further research is needed, especially regarding the experience of people with dementia and their relatives who participated in counseling according to §7a SGB XI. It seems essential to understand the perspective of people with dementia and their relatives to improve and tailor counseling services in Germany.

**Registration:**

The review protocol was prospectively published (BMJ Open 12:e059771, 2022).

**Supplementary Information:**

The online version contains supplementary material available at 10.1186/s12913-023-09155-7.

## Background

Living with dementia and its accompanying symptoms can be challenging for people with dementia and their relatives [2–5]. Care counseling for people with dementia and their relatives offers an opportunity to provide tailored information based on the assessment of individual needs within the context of the progression of dementia. Providing care counseling services can contribute to maintaining and/or improving the individual’s quality of life and reducing the burden on his or her relatives providing care [6–11]. The literature suggests that the need for counseling and that counseling for people with dementia differs from counseling for those without dementia [12–15]. In addition, care counseling in general often plays a key role in psychosocial and psychoeducational interventions for [16, 17] and the perspective and/or experience of people with dementia, their relatives, and professionals on these interventions are a central aspect of dementia care research [18–20].

In Germany, there are various health care insurance agencies (e.g., welfare or private insurance agencies) that offer care counseling according to the various paragraphs of the Social Law, Book XI (SGB XI) [21]. The specific care counseling service (§7a SGB XI) is a voluntary offer that is funded by the welfare and private insurance care agencies (since Jan. 2009). People who have been assessed for the degree of need of care, have a legal right to receive counseling services [22, 23]. The offices for this service are mostly located in local counseling service centers. Counseling is provided by a trained professional (nurse/social worker) who works for a health care insurance agency. This counseling service can includes the following steps: identifying care needs, providing counseling services, developing a care plan, implementing the plan, evaluating and/or adapting the plan if needed, and providing information about services to relieve the burden on relatives [21, 24]. Accordingly, the service can range from a single counseling session on a specific care topic to an comprehensive ongoing counseling service offer [22, 25–27]. There seems to be no general quality monitoring system. So far, only a guideline how to conduct counseling sessions and what kind of personal requirements are needed to become a §7a SGB XI counselor exist [23]. Furthermore, and in comparison, to other countries [28, 29], it seems that at the moment no quality standard for people with dementia and their relatives has been defined [30]. As a result, it remains unknown to what extent this specific counseling service (§7a SGB XI) addresses the needs of people with dementia and their relatives. To our knowledge, there is no systematic overview focusing on studies evaluating this counseling service in Germany for people with dementia and their relatives. To address this gap, we conducted a scoping review with an emphasis on gray literature of (national evaluation) reports and studies of counseling services according to §7a SGB XI [31, 32].

## Research questions

We developed the following three research questions (1,2, and 3) with additional sub questions (a):

1. Which counseling concepts and structures for people with dementia and their relatives have been developed and/or provided since the implementation of §7a SGB XI in Germany?
  - Which concepts and structures are currently being discussed as supportive for those who seek counseling?
2. How does digital support counseling in the context of §7a SGB XI for people with dementia and their relatives?
  - What implications does this have on providing counseling?
3. How do people with dementia and their relatives experience counseling according to §7a SGB XI?
  - What care needs do they articulate during counseling?

## Methods

**Fig. 1:**
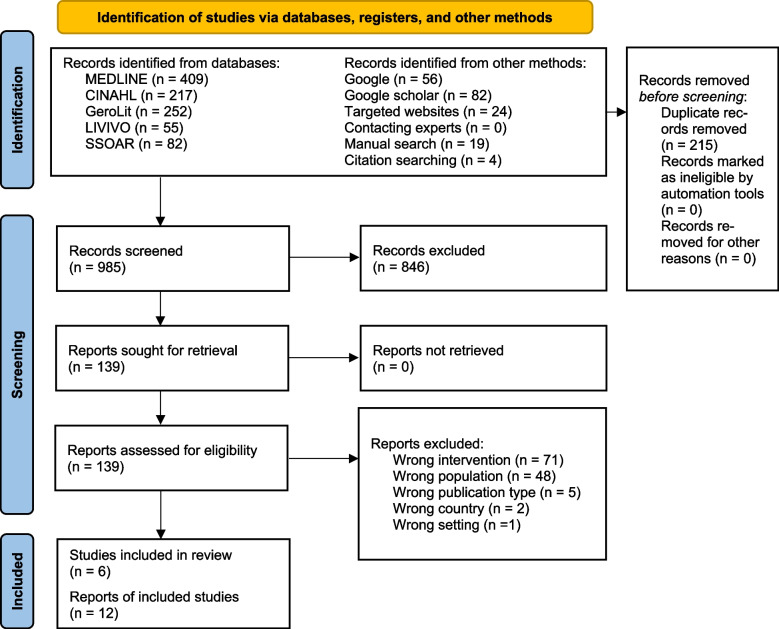
PRISMA 2020 flow diagram [35] demonstrating the identification, screening and eligibility assessments of records preceding scoping review inclusion

We published a review protocol describing our methodological approach [1] in detail. According to the publication and commentary from Pieper, Ge [33], we reused the text of our review protocol for the method section in this publication and made changes where the process differs between the planned and conducted methodological approach. Whenever applicable, we used the Preferred Reporting Items for Systematic reviews and Meta-Analyses extension for Scoping Reviews Checklist [34] and the flow chart of the updated Preferred Reporting Items for Systematic Reviews and Meta-Analyses guidelines [35] to report our scoping review (Supplementary Table 1 and Fig. 1).

### Search strategies

Related to our three research questions and sub questions, our focus was on published studies, analyses, and evaluations of the specific counseling service (§7a SGB XI) implemented in Germany. We therefore focused on gray literature, applied the described approach by Godin, Stapleton [36], and developed a gray literature search plan with an additional strategy for the search in academic electronic databases. This search plan includes the following search strategies: 1) gray literature databases, 2) Google search engines, 3) targeted websites, 4) contacting experts and 5) additional searching in academic electronic databases. All search strategies were conducted from November to December 2021.

#### Strategy 1: Gray literature databases

To identify relevant German electronic databases listing gray literature, we used the descriptions of Nordhausen and Hirt [37]. As a result, we considered the following specific German electronic databases: Livivo, GeroLit (via GBV) and SSOAR (via GESIS). As search strings, we used a simplified form (e.g., focusing on fewer combinations and reducing the search terms) of the search string we created for searching the academic electronic databases (see Strategy 5: Search in academic electronic databases). The search strings for the three different databases can be found in Supplementary Table 2. One researcher conducted these searches (MR-M).

#### Strategy 2: Google search engines

Despite the description of Godin, Stapleton [36], no customizing of the search engines was carried out in the second search strategy. Owing to country-specific factors and the associated technical requirements, we searched Google and Google Scholar using the anonymous function in our web browser (Safari) to ensure that our search was not overly influenced by the individual search history of the reviewer (CM). We defined search strings (Google *n* = 10; Google Scholar *n* = 10) with multiple combinations of search terms (Supplementary Table 3) based on our research questions. The first 10 pages of Google and the first 15 pages of Google Scholar representing approximately 100/150 hits were searched by one reviewer (CM). Findings that at first sight appeared to be related to the research questions and met the inclusion criteria in terms of publication type were included in the next steps of the screening process (see source of evidence selection).

#### Strategy 3: Targeted websites

In accordance with the descriptions of Stansfield, Dickson [38], we considered the inclusion of German websites from (non)-government organizations/institutions, research-active non-government organizations or centers, the National Association of Statutory Health Insurance, providers of counseling services (such as insurance companies, case managers, and care support centers), and community organizations. To identify relevant websites, we first conducted a Google search to identify relevant organizations for this topic [36]. A list of websites was created and supplemented with further websites relevant to the topic (see Supplementary Table 5). Second, one reviewer (DP) hand searched each of the relevant websites for potentially relevant records. Findings that at first view appeared to be related to the research questions and met the inclusion criteria in terms of publication type were included for further screening (see source of evidence selection).

#### Strategy 4: Contacted experts

One reviewer (MR) contacted experts regarding care counseling according to §7a SGB XI in Germany. Experts were recruited from practice partners of the German Center for Neurodegenerative Diseases (Witten site) and were contacted via email with brief project information and with the request to send any potential literature or websites of interest related to the topic. The list of experts with a focus on their profession and occupational activity is reported in Supplementary Table 6.

#### Strategy 5: Search in academic electronic databases

For the additional search in academic electronic databases, we searched the electronic databases MEDLINE (via PubMed) and CINAHL (via EBSCO). Our search terms were derived from our research questions and supplemented with additional free search terms and indexing words from an initial explorative search. These search terms were clustered according to the “PCC” mnemonic and resulted in a search string [32]. The search string was developed by the first reviewers of the review (MRM/CM) and was checked by the two other reviewers (DP/MR) using Peer Review of Electronic Search Strategies (PRESS) [39]. The search string was developed first for MEDLINE (via PubMed) (Supplementary Table 4) by the same researcher mentioned in Strategy 1 and then adopted for CINAHL (via EBSCO) according to RefHunter Version 5.0. [37].

#### Additional citation tracking

For the identified gray literature, we provided backward citation tracking via reference lists. For the identified literature through our academic electronic database searches, we provided backward and forward citation tracking via reference lists and Google Scholar.

### Selection of evidence sources

Records identified through our electronic database searches (strategies 1 & 5) were imported into Covidence [40] and automatically checked for duplicates. Titles and abstracts of records were screened by two reviewers (MRM/CM) independently against the inclusion criteria (Table 1). Full texts were also independently screened for inclusion by the same two reviewers, and reasons for exclusion of full texts were recorded. During the screening process, disagreements between the two reviewers were resolved through a discussion between them. During the screening process, no disagreements between the two reviewers needed to be resolved with the other coauthors (DP/MR).

**Table 1:** Inclusion and exclusion criteria [1]

Criteria	Definition
*Population*	- People with symptoms of dementia (with or without a dementia diagnosis) - Relatives of people with symptoms of dementia (with or without a dementia diagnosis)
*Concept of Interest*	- Counseling according to §7a SGB XI related to the care of people with dementia (with or without a dementia diagnosis) - Counseling about care is not part of the nursing process
*Context*	- Germany
*Types of evidence*	- Focus on gray literature in the form of (evaluation) reports, practice articles and theses - Literature published in peer-reviewed journals
*Other*	- Languages: German and English - Year: no restrictions

For search strategies 2–3, we created an Excel spreadsheet to record the search strategy, including information *on the name of the resource, searcher, date, search string, and number of potentially relevant records* [38]. For strategies 2–4, potentially relevant records were collected in an EndNote Version 20 [41] file stored in a shared NextCloud [42] folder and automatically checked for duplicates at the end of the search process. The title/abstract and full text were screened independently by two reviewers (MRM/CM) in Covidence [40] against the inclusion and exclusion criteria (Table 1). Exclusion reasons for full texts were recorded. Disagreements were discussed between the two reviewers, and no disagreements between the two reviewers needed to be resolved with the other coauthors (DP/MR).

Our inclusion criteria were pilot tested for the first 25 records. No adjustment of the inclusion criteria was required because disagreements between the two reviewers were less than 25% [32].

#### Data extraction

Our data extraction form was based on the template for scoping reviews developed by the Joanna Briggs Institute [43]. We considered the following aspects: *General information* (author, year, primary and additional publication, publication type, aim of the publication), *Study design & methods* (study design, methods), *Participants* (study population, age), and *Results*. Data extraction was performed independently by two researchers (MRM/CM). Disagreements about the extractions were discussed between the two researchers. During the extraction process, no disagreements between the two researchers needed to be resolved by the other coauthors (DP/MR).

#### Presentation of the results

We divided the identified studies into three themes and mapped our extracted data in a table (see Table 2) and descriptively described the results based on our three research questions [43].

**Table 2:** Study characteristics of the included studies

General information	Study design, methods, and participants	Results
**Primary publication:** Bartholomäus, Gruschinski [44] **Additional publication:** Bartholomäus [45], Brandes, Kwirand [46], Bartholomäus, Gruschinski [47] **Publication type:** Report **Aim:** Implementation/Evaluation of the FIDEM (Frühe Informationen und Hilfe bei Demenz) concept	**Study design:** ■ Implementation of the FIDEM concept with evaluation (mixed methods design) **Methods and participants:** ■ Standardized fax system (*n* = no information) ■ Interviews with primary care physicians (*n* = no information) and relatives (*n* = 14) ■ Questionnaires for the evaluation of the workshops (primary care physicians *n* = 55; medical assistants *n* = 117) ■ Questionnaires for the general evaluation (primary care physicians *n* = 20; medical assistants *n* = 17; social service workers *n* = 27)	■ The FIDEM concept consistently focuses on the care of primary care physicians and the referral of patients with dementia to nonmedical support services and offers a structured cross-sectoral approach involving all stakeholders ■ The FIDEM network consists of various actors such as primary care physicians, self-help groups, occupational therapy, low-threshold care and respite services and care counseling ■ The care counseling according to §7a SGB XI is one of the actors in the FIDEM network
**Primary publication:** Beikirch, Braeseke [48] **Additional publication:** No information **Publication type:** Report **Aim:** Analysis of the status quo in the existing care support centers regarding coordination and networking tasks as well as quality assurance	**Study design:** ■ Evaluation **Methods and participants:** ■ Literature research and analysis ■ Nationwide online survey of care support points (*n* = 184) ■ Workshops (*n* = 2) with representatives of providers and employees from care support points in various German states	■ 144 care support points stated that they cooperate with dementia services ■ 79% of the care support points rated this cooperation as good (among the top 3)
**Primary publication:** Braeseke, Delekat [49] **Additional publication:** No information **Publication type:** Report **Aim:** Evaluation of the work of the care support points in the state of Brandenburg as a basis for deriving targeted measures for further development of care counseling according to §7a	**Study design:** ■ Evaluation **Methods and participants:** ■ Focus groups (*n* = 12 staff from 6 care support points, of which *n* = 6 care counselors, *n* = 4 social counselors, *n* = 2 network coordinator) ■ Web-based activity recording (14 days) and analysis (*n* = 50) ■ Online survey of staff at care support points, including branch offices and outreach offices (*n* = 41) ■ Online survey for structural assessment (*n* = 36 care support points) ■ Online survey of regional actors (e.g., actors in the district or city administration, home care services, counseling centers) (*n* = 426) ■ Written survey of the users of the care support points (*n* = 253) ■ Online discussion (*n* = 27, consisting of care counselors, social counselors, other care support point staff, network representatives, administration/county administration/city administration/ministry), health/care insurance fund, disability representative/advisory board/advocate, self-help association)	■ 69.9 % of the care counselors in the counseling service centers are trained for counseling according to §7a SGB XI ■ 38.7 % of the surveyed users (or their relatives) have a need for help and support due to mental disability (e.g., dementia) ■ Desire of the interviewed counselors of the counseling service centers for further training on the topic of dementia ■ Desire of the interviewed counselors of the counseling service centers for further training on the topic of dementia ■ 80 % of the surveyed care support counselors use target group-specific information material ■ Need for more outreach and target group-specific counseling services for people with dementia and their relatives ■ 14.6% of the surveyed counselors in the care support points refer to a (specific trained) colleague within the counseling service center ■ From the point of view of the regional stakeholders, the needs of people with dementia or their relatives can be met by the counseling service centers (41.2% stated yes, 28.2% partly) ■ 43.9% of the surveyed counselors of the counseling service centers stated that they frequently cooperate with dementia counseling centers, 46.3% occasionally, 2.4% usually do not cooperate at all ■ 41.5% of the surveyed counselors of the counseling service centers refer to relevant counseling services (e.g., dementia counseling services, network partners, self-help groups) ■ Improvement of the use of counseling according to §7a is discussed with the linkage of the obligatory counseling visits according to §37 Abs3 SGB XI
**Primary publication:** Dehl, Nolting [50] **Additional publication:** GKV-Spitzenverband [51] **Publication type:** Report **Aim:** Evaluation of care counseling and structures according to § 7a paras. 1-4, 7-8, §7b Ans. 1-2 and §7c SGB XI as well as counseling interventions in the patient's own home according to §37 paras. 3-8 SGB XI within the framework of the legal reporting obligation according to §7a par. 9 SGB XI	**Study design:** ■ Evaluation **Methods and participants:** ■ Nationwide analysis: ■ Secondary data (official nursing care statistics, routine data from the care insurance, data from the Federal Medical Service) ■ Online survey of care insurers (*n* = 37) ■ Survey of insured persons (applicant) (*n* = 2250) ■ Survey of insured persons (care allowance recipients) (*n* = 2676) ■ In-depth regional analysis: ■ Investigation counseling structures ■ Focus groups (*n* = 15; care counselors of the care insurance and service providers of the care insurance, care support points, municipal representatives, representatives of those affected, outpatient and/or inpatient care facilities, hospitals, and other local counseling centers) ■ Online survey of counseling centers (*n* = 99; of which *n* = 39 offer counseling according to §7a SGB XI) ■ Online survey of care counselors (*n* = 294; of which *n* = 262 provide counseling according to §7a SGB XI, *n* = 88 provide counseling according to §37 para. 3 SGB XI) ■ Telephone interviews with users of the counseling service (*n* = 8) ■ Written survey of the users of the counseling service (*n* = 299)	■ Counseling in accordance with §7a SGB XI does not, for the most part, include separate counseling services for people with dementia and their relatives ■ 74.7% (*n* = 29) of the counseling centers offering care counseling according to § 7a SGB XI state that they have dementia expertise ■ 72.0% (*n* = 177) of the counselors surveyed, use (additionally) specially tailored information material for people with dementia and their relatives ■ 35.4% (*n* = 87) of the counselors surveyed refer inquiries from people with dementia and their relatives to specially trained colleagues or to special counseling centers ■ 39.8% (*n* = 98) of the care counselors surveyed have received special advanced training or continuing education in counseling relatives of people with dementia ■ Counseling requests from people with dementia and/or their relatives accounted for 31.6% of the average counseling volume within four weeks ■ In most regions, there are specialized contact points for people with dementia and their relatives, e.g., dementia service centers in their region, regional offices "Aging, Care and Dementia", Alzheimer Society, etc., in addition to the counseling service according to §7a SGB XI ■ From the perspective of the care counselors, cooperation with specialized local services, e.g., with contact points for people with dementia, is rated as good
**Primary publication:** Lobenwein [52] **Additional publication:** No information **Publication type:** Practice article **Aim:** Field report on dementia counseling using the example of the Neuendettelsau Deaconry's specialist center for family caregivers at the Roth Care Support Center	**Study design:** **Methods and participants:** ■ Report of one care counselor	**Overall** ■ Relatives of people with dementia usually take up the offer of counseling only after a “long road" (existence of dementia) ■ People with dementia often take up the offer of counseling in the early stages of their disease **Procedure of contacting and counseling** ■ Contact usually by telephone ■ Counseling appointment at the counseling service center or at home (approx. 90 min) ■ The initial situation is clarified in the first counseling (e.g., symptoms, resources) **Needs and topics of counseling** ■ Dementia and experience ↪ Basic knowledge about and understanding of the disease ↪ Relationship changes ↪ Changing behavior (e.g., self-harm, behavior that challenges the caregivers) ↪ Acceptance of dementia *Burden* ↪ Own helplessness ↪ Solutions and reflection of conflict situations ↪ Interaction with people with dementia *General conditions* ↪ Determining and applying for payments from long-term care insurance ↪ Transition to primary care physician for diagnosis of dementia ↪ Medical treatment ↪ Clarification of the legal situation (e.g., guardianship, health care proxy) *Help and support services* ↪ Acknowledgment that supports services are necessary ↪ Informing and counseling about relief options ↪ Building a relief system
**Primary publication:** Paulicke [53] **Additional publication:** Paulicke, Buhtz [54], Paulicke, Buhtz [55] **Publication type:** Theses/literature published in peer-reviewed journal **Aim:** Identification of information and counseling needs of caregiving family members of people with dementia for the integration of assistive technologies into care and theoretical conceptualization of a transformative understanding of information and counseling	**Study design:** * ■* Sequential-explorative study design * ■* Mixed methods survey **Methods and participants:** * ■ *Focus groups with relatives of people with dementia (*n* = 46) * ■ *Expert interviews with counselors (*n* = 5) * ■ *Survey of counselors (§7a SGB XI) (*n* = 47) using standardized questionnaire	**General aspects of counseling for relatives of people with dementia** * ■ Basic requirements for counseling relatives of people with dementia* ↪ Simple access to counseling ↪ Comprehensible language ↪ Establishing a relationship ↪ Trust as the basis of counseling (especially for older relatives of people with dementia) ↪ Long-term counseling ↪ Fixed contact person ↪ Embedding of counseling in existing services that are regularly used by the relatives of people with dementia * ■ Barriers to counseling relatives of people with dementia* ↪ Low level of awareness: Many family caregivers are generally unaware of the possibility for counseling ↪ Limited time resources among relatives **Digitalization of counseling from the counselors’ point of view** * ■ *Video-based counseling ↪ Counseling via telephone ↪ E-mail exchange ↪ Simple and easy design of digitalization **Assistive technologies as a counseling topic from the counselors’ point of view** * ■ *Counseling on assistive technologies should be an integral part of the counseling process * ■ Forms of mediation of assistive technologies* ↪ Sample integration ↪ Possibility of testing ↪ Handbooks ↪ Outreach counseling at the homes of people with dementia and their relatives * ■ *Younger counselors are described as more suitable for counseling on assistive technologies * ■ Counseling skills* need to be enhanced in relation to assistive technologies **Assistive technologies as a counseling topic from the perspective of relatives of people with dementia** * ■ *Assistive technologies are not perceived as subject for counseling and other information opportunities * ■ *Relatives are more likely to receive information about assistive technologies to support caregiving (e.g., through acquaintances, support groups, pharmacies) by chance * ■ Wishes of the relatives of people with dementia* ↪ Information about assistive technologies in established settings and social gathering places and/or communities (e.g., church congregation, senior afternoons) ↪ Involvement of health insurance companies & municipalities as contact partners * ■ *Counseling & training on assistive technologies ↪ Integration into care and/or dementia counseling ↪ In form of test days, trying out assistive technologies, guidance by nurses

## Results

We identified 1,015 records using the database search. In addition, we were able to identify 185 records through other methods (e.g., Google search, targeted websites). After removing duplicates, we screened 985 records for relevance and included 6 studies published in 12 reports in the review [44, 48–50, 52, 53]. Figure 1 illustrates the identification, screening, and eligibility assessment of records prior to their inclusion in the scoping review.

### Study characteristics

Out of the six included studies, four were reports [evaluations *n* = 4 [48–50]; concept *n* = 1 [44]]. Additionally, we identified one practice article [52] and one doctoral thesis [53]. The publication period ranged from 2012 to 2021, with most publications occurring from 2018 to 2021 (*n* = 4) [48–50, 53]. All evaluation reports were published in 2018 and 2021 [48–50]. The evaluation reports did not directly focus on people with dementia and their relatives but included this population in their overall evaluation. In contrast, the other three reports [44, 52, 53] focused specifically on counseling for people with dementia and their relatives. None of the explicit studies reported the inclusion of people with dementia as study participants [44, 48–50, 52, 53]. Five studies mostly focused on interviewing counselors and other stakeholders [44, 48–50, 52]. In the study by Paulicke [53], focus group interviews were conducted with relatives of people with dementia. The study characteristics of all included studies are provided in Table 2.

#### Summary of the included studies

The results of the included studies were divided into the themes "understanding conceptual dimensions" (Questions 1 and 1a), "digitalization of counseling " (Questions 2 and 2a), and "understanding the perspective of those being counseled" (Questions 3 and 3a).

##### Understanding conceptual dimensions

We did not find any studies that focused on the evaluation of concepts and counseling structures according to §7a SGB XI for people with dementia and their relatives. However, in the included studies, we were able to identify elements of dementia-sensitive counseling services and structures. In addition, we identified information about the professionals, their competencies in counseling people with dementia and their relatives according to §7a SGB XI and their cooperation with other (dementia) counseling services.

In the studies of Braeseke, Delekat [49], Paulicke [53], Lobenwein [52], different structural aspects are described that could be defined as relevant elements of dementia-sensitive counseling services according to §7a SGB IX. These elements are (a) the provision of counseling services (e.g., outreach strategies), (b) initiating care for the person with dementia if the relative seeks counseling services, (c) integrating counseling services into the everyday lives of relatives (e.g., counseling offices located in shopping malls), (d) building longer-term relationships with the counselors, and (e) the use of lay language during counseling.

Information on the structure of counseling services was identified in the study of Dehl, Nolting [50]. They reported that people with dementia and their relatives accounted for a third of all counselings each month according to §7a SGB XI. However, this study did not provide details about dementia-specific counseling services according to §7a SGB XI in the districts or cities investigated in their studies. In general, reference is made to the fact that the counseling service according to §7a SGB XI is mostly unknown to people with dementia and their relatives and/or is not used [53]. To increase awareness, acceptance, and use, the authors [49] recommend implementing §7a SBG XI services in other mandatory counseling structures (§37) for people who receive health care services or personal budget from their care insurance agencies.

Regarding the competencies of professionals and their cooperation with other service providers, the following professional groups provide counseling services (§7a SGB XI): nurses, social insurance employees, social pedagogues/social workers, care/case managers, sociologists, nurse educators and health scientists. Most counselors have a qualification as a nurse (52.2%) or are an employee of a social insurance agency (39.1%) [49, 50]. This is also reported in the publications of Paulicke [53] and Lobenwein [52]. In both publications, the counselors are nurses. In addition, Dehl, Nolting [50] provide details about the competencies and training needs of professionals providing counseling services (§7a SGB XI). Thus, 74.7% of the care counseling service centers indicated having a dementia expert among their staff. However, a survey of these counseling service centers [50] concluded that only 39.8% stated that their staff had attended specific dementia training. Accordingly, the staff members of counseling service centers describe a desire for specific dementia training. In a concrete counseling situation, most target groups receive dementia specific information materials (72–80%) [49, 50]. Beikirch, Braeseke [48], Braeseke, Delekat [49], Dehl, Nolting [50] reported that counseling service centers predominantly cooperate with dementia counseling centers in their region: 35.4–41.5% recommend that people with dementia and their relatives should contact these centers [49, 50]. Overall, cooperation with specialized service centers (dementia counseling centers in most federal states in Germany) is described as good from the perspective of the counseling service centers. One of these specialized dementia counseling centersis listed among the Top 3 best-rated facilities for cooperation [48]. Established (specialist) physicians (20.0%) and primary care physicians (25.0%) received negative rating scores as cooperating partners [48].

Finally, we were able to identify one concept in which counseling according to §7a for people with dementia and their relatives is embedded [44]. In this concept, the primary care physician acts as a gatekeeper and mediator of the various health care services. It is expected that this will increase utilization and improve the care of people with dementia and their relatives living at home.

##### Digitalization of counseling

We could not identify any studies that focused on or evaluated the digitalization of counseling services (§7a SGB XI) for people with dementia and their relatives. Consequently, we were unable to identify any detailed information on the extent to which digitalization affects these counseling services.

In the study by Paulicke [53], methods for digital counseling services according to §7a SGB XI for people with dementia and their relatives are described as promising from the perspective of the counselors. This includes video-supported counseling, counseling via telephone or e-mail exchange between the counselor and the person seeking counseling. The authors point out that digital counseling services need to be simple to use for counselors and the people with dementia and their relatives. Digitalization should not appear as an additional barrier when seeking counseling [53].

##### Understanding the perspective of those being counseled

We could not identify any publications that examined or evaluated target group-specific needs related to people with dementia, nor did we find publications that addressed the perspective of people with dementia and their relatives when seeking counseling (§7a SGB XI). We found one study that described the need for counseling of people with dementia and their relatives from the perspective of a counselor [52]. Lobenwein [52] reports complex needs for counseling among relatives that correspond with the specific symptoms of the family member's dementia. These needs can be divided into the topics "dementia symptoms and experience" (refers to basic knowledge of dementia & understanding of the disease, changes in relationships, changed behavior, e.g., personal hygiene, self-harm, and acceptance of dementia), "burdens" (refers to own helplessness, solutions and reflection of conflict situations, interaction with people with dementia), "framework conditions" (refers to determination and application for benefits from the care insurance, referral to the primary care physician for diagnosis of dementia and medical treatment, legal situation, e.g., guardianship, health care proxy) and "relief" (refers to acknowledgment that support services are needed, identifies respite options, builds a respite system). Furthermore, Lobenwein [52] points out that people with dementia often come for counseling during the early stages of dementia. If this is the case, people with dementia often seek clarification of the disease (e.g., diagnosis, early symptoms of dementia). Regarding the relatives of people with dementia, Lobenwein [52] describes the opposite and reports that they often only seek counseling services after they have already been a caregiver for a long time.

## Discussion

To our knowledge, our “gray-shaded” scoping review is the first systematic overview, which focuses on the concepts, structures, and experiences of people with dementia and their relatives regarding counseling according to §7a SGB XI. The strengths of our review are the methodological quality and the systematic (reproducible), and comprehensive approach to identify gray and peer-reviewed published literature on this specific topic. Our approach to identifying and include gray literature in our review allowed us to obtain a broad national scope of the topic area.

We identified six studies within a total of 12 reports [44, 48–50, 52, 53]. Based on our findings, we defined three themes: "understanding conceptual dimensions", "digitalization of counseling ", and "understanding the perspective of those being counseled". We did not find any evaluation report related to counseling services according to §7a SGB XI with a comprehensive focus on people with dementia and their relatives. Although people with dementia and their relatives make up one-third of the people seeking counseling services (§7a SGB XI), no dementia specific counseling services seem to exist, only one concept embedded a dementia specific counseling service into their primary care structure and processes [44, 48–50].

Furthermore, none of the identified studies presented the experiences from the perspective of people with dementia or interviewed them about their involvement in counseling services in Germany. Mainly the perspective of counselors or other stakeholders were presented in the identified studies [44, 48–50, 52]. Consequently, the perspective of people with dementia and that of their relatives remains mostly unknown regarding counseling services (§7a SGB XI) in Germany. However, understanding the perspective of this particular population appears to be essential since. Lobenwein [52] states that people with dementia and their relatives have different and more comprehensive needs and demands for counseling and counseling structures according to §7a SGB XI than people with non-dementia related care needs. This emphasizes the fact that within the progression of dementia and the accompanying symptoms, people with dementia and their relatives appear to have additional support needs (e.g., specific information about dementia related phenomena) and require specific structural conditions (e.g., support for the person with dementia during counseling sessions) and/or locations (e.g., integrated into everyday activities such as shopping) [52, 53]. These findings are confirmed by the international literature [12–15]. Consequently, it seems crucial to investigate the perspective of the persons with dementia and their relatives, to better understand their needs for counseling services (not only according to §7a SGB XI) and to develop and implement tailored services.

We did not identify any concrete results about initiatives for digitalization [56, 57] of counseling services according to §7a SGB XI for people with dementia and their relatives. However, the literature shows that the implementation of digital structures for counseling services (e.g., telephone and/or e-mail) for relatives has been perceived as a relief and an opportunity to make use of counseling services and lead to a positive evaluation [10, 58, 59]. Consequently, this needs to be considered when improving counseling services for people with dementia and their relatives [30, 60].

Furthermore, we need to contemplate how awareness and acceptance of counseling services (§7a SGB XI) can be increased. Usually, the primary physician is the first point of contact or the first contact with the health care system for people with dementia and their relatives [61]. Bartholomäus, Gruschinski [44] recommends that a primary physician can act as the primary contact person for the referral to the counseling service centers and as a gatekeeper within the health care system, which may help improve awareness and the use of counseling services according to §7a SGB XI among people with dementia and their relatives.

Finally, it can be assumed that counseling people with dementia requires different skills of the counselors and a variety of methods to provide counseling to people with dementia and their relatives [62]. To increase these skills among counselors, it seems necessary to develop and implement specific training and education programs that consider the needs of people with dementia and their relatives. This could improve the awareness and knowledge of counselors and their interactions with people with dementia and their relatives in counseling and contribute to better patient-reported outcomes [63].

## Limitations

Our gray-shaded scoping review has some limitations. First, we only included studies that explicitly addressed counseling for people with dementia and their relatives according to §7a SGB XI. Consequently, we did not include studies if it was not clear whether they referred to this type of counseling and this population. This may have resulted in not identifying all relevant publications for our review. Second, it remains unknown to what extent evaluation reports on counseling services are published (open access), since evaluation results are not always published in detail, but primarily available to political stakeholders. Consequently, publication bias cannot be excluded in our review. Last, despite the use of the anonymous function of the browser (Safari) used for the Google searches, this appears to be difficult to reproduce, since personal characteristics (search history) probably nevertheless caused an adjusted result in Google and Google Scholar.

## Conclusion

We identified a few gaps in the provision of counseling services for people with dementia and their relatives: one-third of the people who used counseling according to §7a SGB XI are people with dementia and their relatives; therefore, we deem it essential (1) to conduct specific evaluations related to this particular population and (2) to understand the perspective of people with dementia and their relatives (for example, how they experience this service). Furthermore, to develop a quality standard [30], a scoping review of counseling services, concepts, and structures for people with dementia and their relatives (beyond §7a SGB XI) is needed to define a substantial evidence base. This could be accomplished by first reviewing the international literature to clarify heterogenous definitions on care counseling for people with dementia in different countries in comparison of how counseling is embedded in case/care management approaches [27]. Furthermore, the development of a quality standard [30] could be done in a participatory manner by involving relevant stakeholders and people with dementia and their relatives and thus include individual preferences of people with dementia and their relatives [64]. This approach appears to be in line with the national dementia strategy in Germany [30] and would support the overall idea of the German dementia strategy to make health care systems more dementia-friendly [65].

## Supplementary Information

**Additional file 1.**

## Data Availability

All data generated or analyzed during this study are included in this published article and its supplementary information files.
